# Drought and Subsequent Soil Flooding Affect the Growth and Metabolism of Savoy Cabbage

**DOI:** 10.3390/ijms222413307

**Published:** 2021-12-10

**Authors:** Alessa Barber, Caroline Müller

**Affiliations:** Chemical Ecology, Bielefeld University, Universitätsstr. 25, 33615 Bielefeld, Germany; alessabarber@t-online.de

**Keywords:** Brassicaceae, *Brassica oleracea* convar. *capitata* var. *sabauda*, climate change, crop plant, drought, glucosinolates, metabolism, soil flooding

## Abstract

An important factor of current climate change is water availability, with both droughts and flooding becoming more frequent. Effects of individual stresses on plant traits are well studied, although less is known about the impacts of sequences of different stresses. We used savoy cabbage to study the consequences of control conditions (well-watered) versus continuous drought versus drought followed by soil flooding and a potential recovery phase on shoot growth and leaf metabolism. Under continuous drought, plants produced less than half of the shoot biomass compared to controls, but had a >20% higher water use efficiency. In the soil flooding treatment, plants exhibited the poorest growth performance, particularly after the “recovery” phase. The carbon-to-nitrogen ratio was at least twice as high, whereas amino acid concentrations were lowest in leaves of controls compared to stressed plants. Some glucosinolates, characteristic metabolites of Brassicales, showed lower concentrations, especially in plants of the flooding treatment. Stress-specific investment into different amino acids, many of them acting as osmolytes, as well as glucosinolates, indicate that these metabolites play distinct roles in the responses of plants to different water availability conditions. To reduce losses in crop production, we need to understand plant responses to dynamic climate change scenarios.

## 1. Introduction

Contemporary global change involves drastic changes in the world’s climate, with more extreme events, such as warmer temperatures, more severe drought periods but also more severe flooding [1,2,3]. Water availability is of high relevance for plant growth, performance and metabolism [4,5]. Under drought stress, stomata close, the net carbon dioxide assimilation rate is thus reduced, and growth is suppressed [5,6]. In contrast, in flooded soil, oxygen becomes depleted, generating hypoxic stress, leading to root damage, and consequently, reduced plant productivity [7]. Similarly as under drought, under flooded soils, photosynthetic activity and transpiration are reduced due to stomatal closure [8]. Subsequent phases with more regular water availability may lead to recovery if damage is not too severe [9]. Responses of plants to individual stresses have been investigated in detail, particularly in crop plants [10,11]. In contrast, fewer studies have focused on the effects of combinations of drought and flooding on photosynthesis, plant hormones and yield [12,13], and particularly little is known about the responses to different sequences of water stresses and potential relaxation from stress on other (metabolic) traits.

Under drought stress, some plants produce smaller but thicker leaves, resulting in a reduced specific leaf area [6], i.e., an increased specific leaf mass. In other plant species or cultivars, soil flooding can likewise cause the production of smaller leaves [14]. Limited or modified growth and productivity in crop plants result from various physiological changes. Drought-stressed plants exhibit reduced water content, with dehydration becoming more severe the longer the stress lasts [15]. Under flooding, water content likewise becomes reduced due to a lower water potential, loss of turgor pressure, reduced oxygen and nutrient availability to roots, and potential changes in the microbial environment [7,16]. Thus, contrasting stresses such as drought and flooding can ultimately lead to similar plant responses, but sometimes via slightly different mechanisms. However, other responses clearly differ, such as the water use efficiency. For example, with mild drought stress, the intrinsic water use efficiency (WUE_intrinsic_; CO_2_ assimilation rate divided by stomatal conductance or transpiration rate) increases, because stomatal closure suppresses water loss more than photosynthesis [6,17]. Likewise, the applied WUE (WUE_applied_; dry aboveground biomass divided by total water availability) can be higher in drought-stressed compared to well-watered plants, as shown in wheat [18]. In contrast, under waterlogging, WUE (calculated as biomass produced per unit water transpired) was observed to be significantly reduced in several millet species, whereas in response to drought, WUE changed in different directions, depending on the species [19]. These results highlight that plants exhibit species-specific responses in adjustments to different types of water stress, as reported previously [20,21].

Water availability also influences the nutrient uptake and metabolism of plants, being limited in dry soils [4,6]. However, effects differ between climatic regions. For example, the carbon-to-nitrogen (C:N) ratio tends to increase in semi-arid environments in response to drought, whereas it decreases in wet temperate ecosystems [22]. Waterlogging leads to reduced total N contents, and thus an enhanced C:N ratio [7,23]. Amino acids play a key role in tolerance to abiotic stress by acting as C or N reserves and as osmolytes [24]. Total amino acid concentrations increased in potato tubers of different cultivars under drought, but decreased under waterlogging [25], whereas species-specific changes were found in total amino acid concentrations of aboveground biomass in different *Brassica* species in response to flooding [26]. The amino acid proline is particularly important in responses to abiotic stress, acting as an osmoprotectant and osmoregulator and scavenging reactive oxygen species produced under drought stress [24,27].

Moreover, several amino acids are important precursors of specialized (secondary) metabolites such as glucosinolates, which occur in all species of Brassicales [28]. Glucosinolates are based on an *S-β*-D-glucopyrano unit linked to an *O*-sulfated (*Z*)-thiohydroximate function. Their metabolism is expensive [29]. The thioglucosidic bond is readily hydrolyzed by myrosinases which, after spontaneous rearrangement, lead to the formation of toxic isothiocyanates, nitriles or other hydrolysis products [28]. Abiotic stresses such as drought or waterlogging result in imbalances in plant metabolism and altered phytohormone signaling [30,31,32]. Directly depending on these factors, it is not surprising that changes in water availability have also been shown to result in either increases or decreases in total and individual glucosinolate concentrations, with responses differing between Brassicaceae species and cultivars and depending on the applied water stress [33,34]. The magnitude of changes in glucosinolate concentrations also varies with the stage of development, as shown, for example, in different *Brassica carinata* varieties [35].

**Figure 1:**
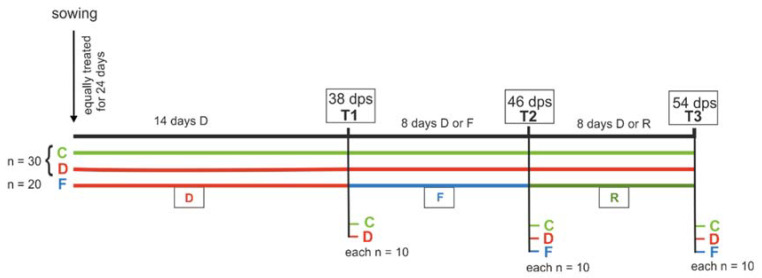
Timeline of the experimental set-up and the different treatments. All plants were treated equally for 24 days and then divided into three groups: control (C, green, *n* = 30), drought stress (D, red, *n* = 30) and soil flooding treatment (F, blue, *n* = 20). Drought-stressed plants were watered less from 24 days post-sowing (dps) onwards; soil-flooded plants were drought-stressed until 38 dps (T1), then soil-flooded until 46 dps (T2) and then not watered until 54 dps (T3) for a potential recovery phase (R).

To test the effects of different water stress regimes on plant growth and metabolism, plants of savoy cabbage (*Brassica oleracea* convar. *capitata* var. *sabauda*) were either kept well-watered (control treatment), grown under reduced water availability and thus continuous drought stress (drought treatment), or first drought-stressed (until T1: 38 days post sowing, dps) then flooded above soil level (partial submergence according to [36]; until T2: 46 dps) and finally not watered any more to allow for potential recovery (until T3: 54 dps) (sequential stress, for simplicity, hereafter called “soil flooding” treatment) (Figure 1). Subsets of plants were harvested at each of the three time points, T1–T3, and shoot biomass, water content, specific leaf mass and applied water use efficiency were measured. Furthermore, leaves were harvested, and carbon and nitrogen contents, as well as amino acid and glucosinolate concentrations and compositions, were measured. Both stress treatments led to significantly suppressed growth, with flooding also reducing the water content of the leaves. Furthermore, the stress treatments had significant and distinct imprints on the metabolic composition of the plants, indicating fine-tuned physiological responses of plants to different types of water stress. Our findings have important implications for crop production under current and future climate change scenarios.

## 2. Results

### 2.1. Growth, Water Content, Specific Leaf Mass and WUE_applied_ of Plants Exposed to Different Water Stress Treatments

**Figure 2:**
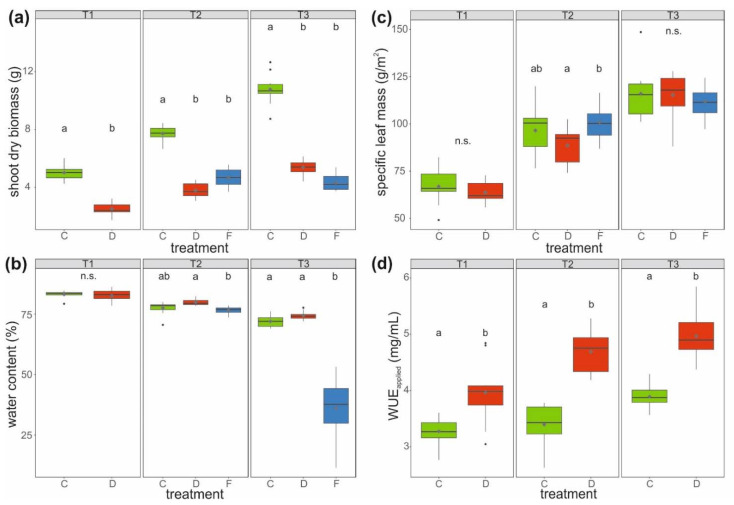
(**a**) Shoot dry biomass, (**b**) water content of aboveground biomass, (**c**) specific leaf mass, (**d**) and applied water use efficiency (WUE_applied_) of savoy cabbage (*Brassica oleracea* convar. *capitata* var. *sabauda*) under different treatments and at three harvest time points (T1, T2, and T3). C: control, D: drought-stressed (for the whole duration of the experiment), F: soil-flooded plants (drought stress with subsequent soil flooding, followed by a potential recovery phase). Data are presented as box-and-whisker plots with median (horizontal line), mean (diamond), interquartile range (box) and whiskers extending to the maximum and minimum values within the 1.5-fold interquartile range; outliers are shown as black dots. Different letters indicate significant differences (*p* < 0.05) among treatments within each harvest time point [T1: (**a**) Mann–Whitney *U*-test, (**b**–**d**) *t*-test; T2 and T3: (**a**,**b**) Kruskal–Wallis-test, (**c**) univariate ANOVA, (**d**) *t*-test; *n* = 10 per treatment and harvest time point], n.s.: not significant.

The aboveground (shoot) dry biomass of well-watered control (C) plants was about twice as high, and thus significantly higher at all three harvest time points compared to that of drought-stressed plants (D) and plants first drought-stressed and then soil-flooded (F). After relaxation from soil flooding, the aboveground biomass of soil-flooded plants was lowest, with only about 40% of that of control plants (treatment F, T3). Although biomass increased over harvest time points, it decreased at T3 in plants in the soil-flooded treatment (Figure 2a).

The water contents of the shoot biomass were comparable (around 83%) between control and drought-stressed plants at T1. At T2, the water content was significantly higher by 3.3% on average in drought-stressed compared to soil-flooded plants, whereas control plants exhibited an intermediate water content (average: 77.4%, Figure 2b). At T3, plants that had been soil-flooded had with <50% a very low water content, with many leaves showing strong signs of withering, whereas control and drought-stressed plants had similarly high water contents (average: 73.2%).

The specific leaf mass did not differ between plants of different treatments at T1 and T3. In contrast, at T2, leaves of drought-stressed plants had a significantly lower (on average 12%) specific leaf mass than soil-flooded plants (Figure 2c).

The WUE_applied_ (dry shoot biomass divided by cumulative irrigation amount of plant individual) was significantly higher (>20%) in drought-stressed compared to control plants at all three time points (Figure 2d). For soil-flooded plants, WUE_applied_ could not be calculated due to the surplus water availability.

### 2.2. C:N Ratio, Amino Acids and Glucosinolates in the Leaves of Plants Exposed to Different Water Stress Treatments

**Figure 3:**
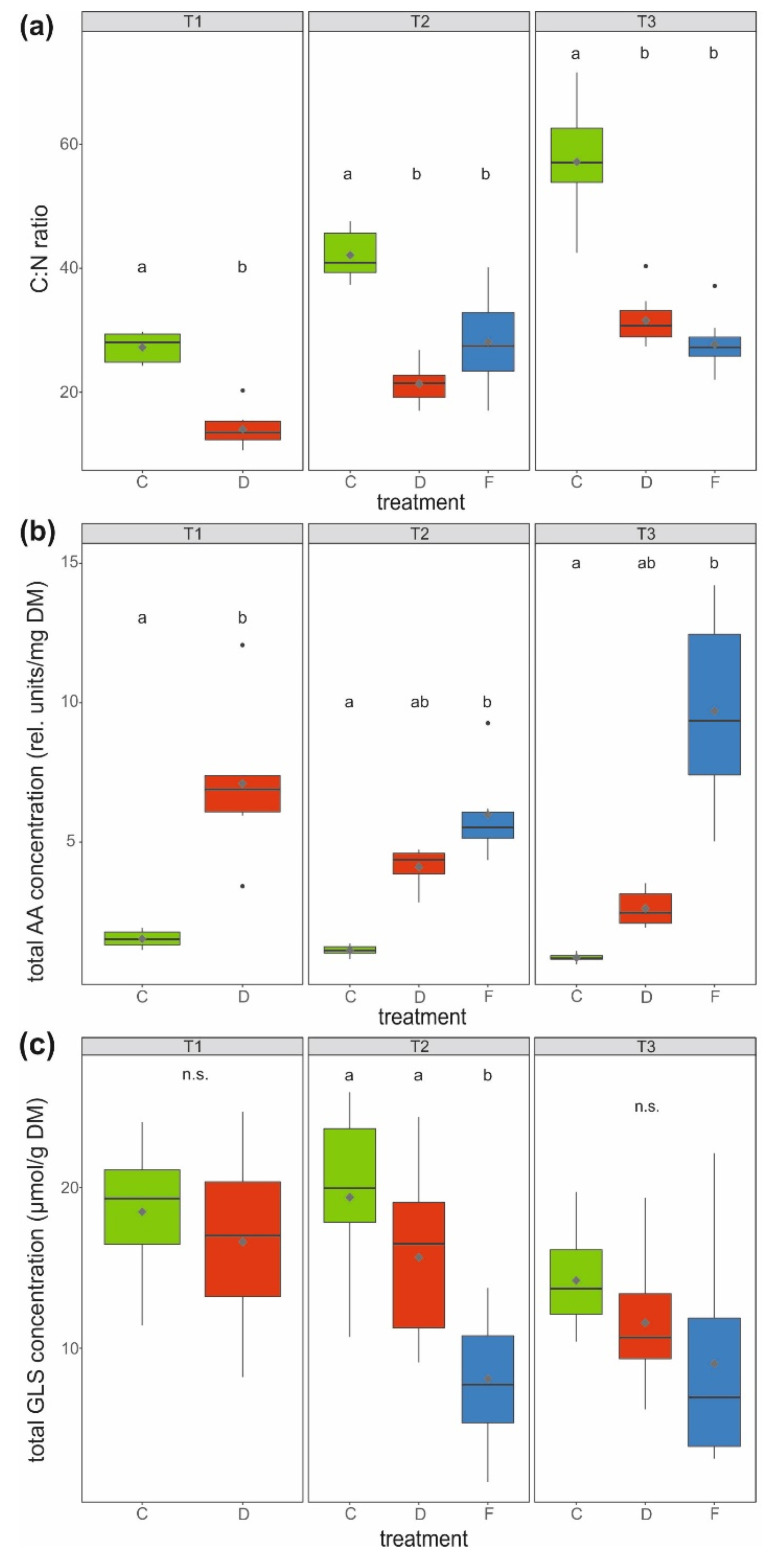
(**a**) C:N ratio, (**b**) total amino acid (AA) concentration (rel.—relative, DM—dry mass; sum of 19 amino acids) and (**c**) total glucosinolate (GLS) concentration (sum of eight glucosinolates) in the leaves of savoy cabbage (*Brassica oleracea* convar. *capitata* var. *sabauda*) under different treatments and at three harvest time points (T1, T2, T3). C: control, D: drought-stressed (for the whole duration of the experiment), F: soil-flooded plants (drought stress with subsequent soil flooding, followed by a potential recovery phase). Data are presented as box-and-whisker plots with median (horizontal line), mean (diamond), interquartile ranges (box) and whiskers extending to the maximum and minimum values within the 1.5-fold interquartile range; outliers are shown as black dots. Different letters indicate significant differences (*p* < 0.05) among treatments within each harvest time point [T1 (**a**,**b**): Mann–Whitney *U*-test, (**c**) *t*-test; T2 and T3; (**a**,**b**) Kruskal–Wallis-test, (**c**) univariate ANOVA; (**a**,**c**) *n* = 10, (**b**) *n* = 6 per treatment and harvest time point], n.s.: not significant.

The C:N ratio was at least twice as high and thus significantly higher in leaves of control compared to water-stressed plants at all measured time points (Figure 3a). In accordance, the total amino acid concentration (sum of 19 amino acids, Table 1) was lowest in leaves of these control plants. At T1, drought-stressed plants had a significantly higher (4.7 times) total leaf amino acid concentration than control plants. At T2 and T3, soil-flooded plants showed significantly higher (11.4 times) total amino acid concentrations in leaves than the control plants, whereas the levels in leaves of drought-stressed plants were somewhat intermediate (Figure 3b).

The total glucosinolate concentration (sum of eight glucosinolates, Table 1) in leaves only differed significantly at T2, with control and drought-stressed plants having, on average, about twice as high concentrations as soil-flooded plants (Figure 3c).

**Figure 4:**
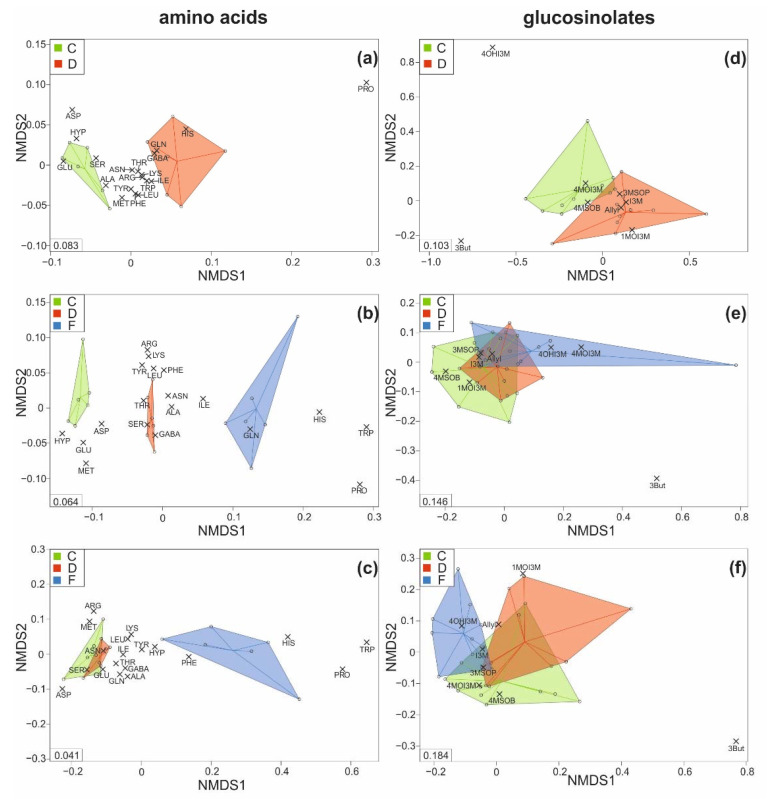
Metabolic patterns of the (**a**–**c**) amino acid and (**d**–**f**) glucosinolate concentrations found in the leaves of savoy cabbage (*Brassica oleracea* convar. *capitata* var. *sabauda*) of C: control, D: drought-stressed (for the whole duration of the experiment), F: soil-flooded plants (drought stress with subsequent soil flooding, followed by a potential recovery phase) at the first (**a**,**d**), second (**b**,**e**) and third (**c**,**f**) harvest time point. Data are presented as non-metric multidimensional scaling plots (NMDS; with Kulczinsky distance) with scores (colored circles; samples within each group are surrounded by convex hulls and all data points are connected to the corresponding medians of the groups) and loadings (grey crosses), full names of the metabolites are given in Table 1. Stress values are given in the left corner of each NMDS. Amino acids: *n* = 6, glucosinolates: *n* = 10 replicates per treatment and harvest time point.

As indicated by non-metric multidimensional scaling, the composition of amino acids was quite distinct between leaves of control and those of drought-stressed plants at T1 (Figure 4a) and between leaves of all three treatment groups at T2 (Figure 4b), as visible by the clear separation of all plant samples belonging to one treatment group. At T3, the leaf amino acid composition of control and drought-stressed plants was overlapping, whereas soil-flooded plants still showed a distinct composition (Figure 4c), with histidine, tryptophan and proline being particularly high in the latter.

The composition of the leaf glucosinolates was only slightly overlapping between control and drought-stressed plants at T1, with 4-methoxyindol-3-ylmethyl glucosinolate and but-3-enyl glucosinolate differing the most, as indicated by the position of the loadings (grey crosses in Figure 4d). At T2 and T3, the composition of the leaf glucosinolates was overlapping among plants of all three treatments, with but-3-enyl glucosinolate again differing the most (Figure 4e,f).

**Figure 5:**
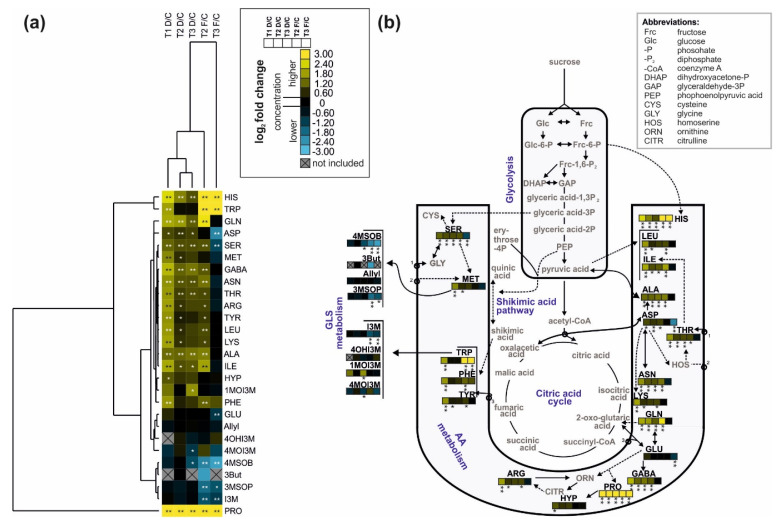
(**a**) Heatmap (average linkage hierarchical clustering with a Euclidian correlation distance matrix) of the amino acids (AAs) and glucosinolates (GLSs) identified in the leaves of savoy cabbage (*Brassica oleracea* convar. *capitata* var. *sabauda*) of C: control, D: drought-stressed (for the whole duration of the experiment) and F: soil-flooded plants (drought stress with subsequent soil flooding, followed by a potential recovery phase) at the three harvest time points (T1, T2, and T3). A higher concentration of a metabolite in a stress group relative to the corresponding control plants is shown in yellow, a lower concentration is shown in blue. Grey and crossed-out boxes indicate metabolites that were not present in at least 50% of the replicates in that group, asterisks show significant differences between the groups (* *p* < 0.05, ** *p* < 0.01, Mann–Whitney *U*-test, *n* = 6). Abbreviations are given in Table 1; (**b**) metabolic pathway map of the identified metabolites (black font), with the changes in metabolite concentration in the different treatment groups compared to the control group depicted in the color-coded heatmap stripes (order of stripes indicated in insert in the center top). Dashed lines indicate intermediates. Additional abbreviations are given in the top right box. (**b**) is based on the metabolic map designed by R. Schweiger [37,38].

In a heatmap, the drought-stressed plants at the three time points were clustered together according to their leaf amino acid and glucosinolate profiles of the leaves, while soil-flooded plants at T2 and T3 were clustered separately (Figure 5a, top of heat map). Most amino acids were modulated [highly significant difference between the stress treatment and control (*p* < 0.01) and fold changes of <0.67 (−0.58 on log_2_ scale) or >1.5 (>0.58 on log_2_ scale)], with enhanced concentrations in the stress treatment groups compared to the control. In contrast, several amino acids were not modulated in soil-flooded plants at T3, and some amino acids (asparagine, glutamine and serine) were even lower in pool size compared to control plants (indicated in blue). Proline was drastically enhanced in pool size in all stressed plants at all time points (indicated by yellow), forming its own cluster (cluster at left side of heat map). Most glucosinolates were not modulated (dark color), although some were lower in pool size in the leaves of soil-flooded plants (Figure 5a).

The glucosinolates derived from methionine, 3-(methylsulfinyl)propyl glucosinolate and 4-(methylsulfinyl)butyl glucosinolate, were only modulated in the leaves of soil-flooded plants, with lower concentrations compared to the control plants, whereas they were not modulated by drought stress (Figure 5b). In comparison, methionine itself was modulated exclusively in the leaves of drought-stressed plants at T1, being higher than in controls. Regarding glucosinolates with indolic side chains, which are derived from tryptophan, only indol-3-ylmethyl glucosinolate was modulated, being lower in the leaves of soil-flooded plants compared to controls. In comparison, tryptophan was modulated in the leaves of drought-stresses plants at T1 and of soil-flooded plants, being mostly higher in concentration in stressed compared to the control plants. Similarly as tryptophan, other amino acids derived from the shikimic acid pathway, such as phenylalanine and tyrosine, were particularly higher at T1 in the leaves of drought-stressed plants as compared with controls. Proline was modulated in the leaves of all stressed plants, whereas hydroxyproline derived from proline did not show any modulation (Figure 5b).

## 3. Discussion

The different water stress treatments had treatment-specific effects on the plant growth and metabolism of savoy cabbage. Most striking were the effects of the potential “recovery” phase on the previously soil-flooded plants, which was revealed to actually be very stressful for the plants.

### 3.1. Effects of Water Stress on Biomass and Water Content

Throughout the experiment, the well-watered control plants produced more biomass than plants of the other treatments, indicating that the latter were indeed stressed. In general, drought stress leads to suppressed growth due to stomatal closure, and consequently, reduced photosynthetic activity and limited nutrient uptake [5,39]. A reduced growth of savoy cabbage plants has also been reported in response to salt stress [40], which acts similar as drought stress. Interestingly, the shoot water content of the drought-stressed plants was not reduced compared to the control plants. This may be explained by a lower transpiration due to stomatal closure in drought-stressed plants [5,6]. In four other *Brassica* species, both a lower biomass production and a lower water content were found in *B. carinata*, the most drought-sensitive species, whereas *B. napus*, *B. campestris* and *B. juncea* exhibited a lower biomass after repeated drought cycles but no change in water content [41], as observed in the present study. With several physiological adaptations (closure of stomata, osmotic adjustments, etc.), plants can prevent water loss during drought, but at the expense of biomass production.

Regarding flooding, already a partial waterlogging can reduce the photosynthesis of mesophyll cells, lower respiration and thus ion uptake and transport via the roots, eventually leading to wilting [7]. In soil-flooded oilseed rape (*Brassica napus*), the photosynthetic rate, stomatal conductance and transpiration rate were reduced, but increased again in a subsequent recovery phase [42]. In the present experiment, the phase after soil flooding without any further addition of water (potential “recovery” phase) was rather detrimental, leading to a very low water content in the leaves and thus wilting. Very likely, roots died, and plant metabolism may have been pushed below the basal metabolic rate, as is often observed under waterlogging or flooding conditions [7]. The damage may either already have been imposed under the oxygen-deficient conditions during flooding and/or oxidative damage experienced when oxygen again reached the roots [43] after taking the pots out of the water. Low oxygen concentrations affect gene expression profiles, plant metabolism, hormonal status, as well as mitochondria and plastids [44,45], whereas negative impacts of waterlogging on mitochondria further weaken photosynthesis [46]. After reoxygenation, reactive oxygen species can accumulate rapidly [47], and cell membrane damage becomes obvious [48], as shown in *Arabidopsis thaliana*. The ability to maintain photosynthesis and membrane integrity highly correlates with the tolerance of plants to waterlogging, differing between cultivars [7,46]. Moreover, the stress responses highly depend on the ontogenetic stage, at which stress is experienced, as well as the duration of waterlogging or flooding. Waterlogging reduced the yield of oilseed rape more severely when applied in a younger than older growth state [49]. With longer periods of soil flooding (2 to 6 days), water content decreased in pigeonpea (*Cajanus cajan*, Fabaceae) and plants of one of two tested genotypes could not recover anymore [9], similarly as observed in the present study in savoy cabbage after an 8-day flooding period. In face of a growing per capita use of vegetables, including cabbages [50], yield losses due to water shortage or flooding should be prevented. Molecular and systems approaches are thus needed to identify mechanisms how to improve crop tolerance to changing climates [51].

### 3.2. Effects of Water Stress on Specific Leaf Mass and WUE_applied_

The specific leaf mass was little affected, only being significantly lower at T2 in drought-stressed savoy cabbage plants. Usually, under drought stress, smaller and thicker leaves are produced [6], which could lead to an increased specific leaf mass. For example, leaves of *B. carinata* showed a significantly lower area, width and length of leaves in plants drought-stressed for 15 days compared to controls [15]. Likewise, under soil flooding, leaf size was found to be reduced in four *Brassica* species, mostly in *B. campestris* [26]. We did not measure the size of the leaves; however, our results may indicate only an intermittent differential investment in leaf mass per area. A slightly lower specific leaf mass may make leaves easier to consume for herbivores [52], because this measure is considered a defense trait, playing an important role in plant–herbivore interactions.

The WUE_applied_ was continuously higher in drought-stressed compared to control plants, meaning that plants under drought stress could use the water more effectively for plant biomass production. This stress adaptation is highly relevant, particularly in crop plants that grow in water-limited agricultural systems [18]. A higher WUE_applied_ under different drought regimes has also been revealed, for example, in wheat [18] and oilseed rape [53]. Different factors could lead to an enhanced WUE_applied_: drought-induced stomatal closure may restrict transpiration before inhibiting photosynthesis, drought may affect the growth rate of plants, and root systems with drought-induced architecture changes may take up water more effectively from dry soils [18,54,55].

### 3.3. Effects of Water Stress on the C:N Ratio, Amino Acids and Glucosinolates in Leaves

The leaf C:N ratio was much higher in the control plants compared to the water-stressed plants, whereas there were no differences among the plants of the two water stress treatments. In dry soils, usually less N is available, and plants have a reduced nutrient uptake [4,6], which results in an enhanced C:N ratio, as found, for example, in Mediterranean shrubs and trees as well as plants of temperate heathlands [22]. However, some plant species of wet temperate ecosystems were found to show the opposite, because they may increase N-uptake but reduce investment in biomass at the same time [22], resulting in a reduced C:N ratio, as also found in savoy cabbage in the present study. Plants grew less under drought; therefore, they may have been able to take up relatively more N, which is only possible if the soil is not too dry. In contrast, seedlings of *B. napus* which were not watered for 10 days showed an enhanced C:N ratio [56]. Likewise, under flooding conditions, plants usually exhibit an increased C:N ratio. However, in our experiment, plants of the soil flooding treatment were initially drought-stressed, which probably mostly shaped the C:N ratio of these plants.

Along with higher relative N contents in the leaves of the stressed plants, these plants showed higher total leaf amino acid concentrations, which could act as N (or C) reserves [24]. Proline is of prime importance in stress tolerance and quickly accumulates both under drought and flooding stress, thereby mediating cellular osmotic adjustments to maintain cellular–water relationships [7,24]. It also has different protective effects for photosystems, for example, by increasing levels of antioxidative compounds [24,57]. Accordingly, proline concentrations, and, in soil-flooded plants, likewise hydroxyproline, which is derived from proline, were enhanced in the leaves of all plants of the stress treatments, contributing to a large degree to the increase in total amino acid concentrations. An increase in proline has been found in seeds, tubers, leaves and in leaf phloem exudates of different plant species in response to drought stress [25,58,59]. The glutamate pathway may be the predominant synthesis route of proline during water stress [60], but reduced proline catabolism could be an alternative explanation [61,62]. In response to flooding, different proline concentrations were found, with no changes compared to control plants [14], or even reductions [25,63]. As discussed for the C:N ratio above, in the present experiment, the drought period preceding the soil flooding phase may have been crucial in the induction of proline in savoy cabbage.

Histidine and tryptophane were particularly highly increased in pool sizes in soil-flooded plants at T2 and T3. Histidine plays an important role in the pH-dependent regulation of aquaporins, which are important to maintain homeostasis [64,65]. Tryptophan is a precursor of melatonin, which acts as a phytohormone and protects plants from oxidative stress, especially water stress [66]. Other amino acids, such as asparagine, serine and glutamine, were reduced in the leaves of savoy cabbage, particularly in soil-flooded plants after the potential recovery phase (T3). Under re-aeration (anoxia-reoxygenation), different metabolic adjustments are necessary to reach the normal homeostatic state [67]. Lower concentrations of certain amino acids may, for example, indicate ineffective tricarboxylic acid cycle replenishment, a disturbed carbohydrate metabolism and/or a delayed energy regeneration during this phase [67].

Although tryptophan concentrations increased in the leaves of soil-flooded plants, indolic glucosinolates derived from this amino acid were not modulated in the present experiment. In contrast, methionine-derived glucosinolates showed lower concentrations compared to control plants, which was not mirrored in methionine. This highlights the very specific regulation of certain metabolites under stress rather than the regulation of entire biosynthetic pathways. Glucosinolates were less modulated than amino acids in savoy cabbage; therefore, they may also be less relevant for stress tolerance against abiotic stresses. Total glucosinolate concentrations only differed significantly at T2, being lowest in soil-flooded plants. To save resources for other metabolites more important in abiotic stress tolerance, the biosynthesis of glucosinolates may be reduced. In broccoli plants (*B. oleracea* var. *italica*), the lowest glucosinolate concentrations were found in drought-stressed plants, and intermediate levels in water-logged plants [68]. Overall, different responses were revealed with regard to glucosinolate concentrations in response to water stress in different species [33,34]. A changed resource allocation in response to changing climatic conditions that modulates concentrations of glucosinolates thus also influences the chemical defense against antagonists [33,69]. Moreover, due to crosstalk between phytohormone signaling, water stress can modify the induction of glucosinolates in response to herbivore attack [68] and alter the interaction between herbivores feeding on roots versus shoots [70].

In conclusion, continuous drought or drought followed by soil flooding were found to have large effects on plant biomass, C:N and different metabolites of the crop species savoy cabbage, which has, to the best of our knowledge, not been investigated before. A potential recovery phase could not reverse the stress effects of the waterlogging, but instead, even enhanced detrimental effects, highlighting that reoxygenation may be particularly stressful. Whether a recovery may be possible after a shorter soil flooding phase remains to be investigated. Our study revealed that differences in water availability can affect structural defenses (e.g., specific leaf mass) and chemical defenses (e.g., glucosinolates) in different directions, which has important implications on the interactions of plants with herbivores or other antagonists. This indicates that under changing climatic conditions in the field, growth inhibition may not only result from too low or too high water availability, but potentially also from changes in the abundance of pests.

## 4. Materials and Methods

### 4.1. Plant Cultivation, Water Stress Treatments and Harvest

Seeds of savoy cabbage (cultivar Vertus 2; Kiepenkerl, Bruno Nebelung GmbH, Everswinkel, Germany) were germinated in the dark in a 1:2 mixture of river sand and soil (Fruhstorfer soil, type P; Hawita Gruppe GmbH, Vechta, Germany), steamed at >90 °C. Seedlings were individually transferred to 2 L pots (11.3 × 11.3 × 21.5 cm) (*n* = 80) placed on dishes to prevent substrate loss but ensure drainage. Seedlings were watered to a soil water content (SWC, mass of water per dry mass of substrate) of 44.8% once, and then to an SWC of 44.1%. The SWC was determined gravimetrically by drying eight subsamples according to DIN 11565 [71]. Plants were grown in a climate chamber at a temperature of 20 °C, relative humidity of 60% and a 16:8 light:dark cycle (photosynthetic active radiation of ~270 µE/m^2^). At 24 days post-sowing (dps), plants were divided into three water stress treatment groups (Figure 1). Control plants (C, *n* = 30) were kept well-watered (SWC about 44%), whereas drought-stressed plants (D, *n* = 30) were watered less (SWC about 15%) until the end of the experiment. Plants of the soil flooding treatment (F, *n* = 20) were first drought-stressed (SWC about 15%) until 38 dps (T1), then flooded until 46 dps (T2) and subsequently not watered any more to allow a potential recovery phase until 54 dps (T3). All plants were watered every other day with tap water (except during recovery) and their position was randomized by rotation. From 38 dps until 46 dps, all pots were placed in transparent 1-gallon (5 L) water containers (Purania Stilles Quellwasser; TSI Consumer Goods, Zeven, Germany). Containers with plants of the flooding treatment were filled with water up to 2 cm above the rim of their pots. At 46 dps, pots were removed and placed back on their dishes. At 35 and 51 dps, all plants received 10 mL fertilizer solution [2 mL fertilizer (Wuxal-Super, 8% N, 8% P_2_O_5_, 6% K_2_O; Hauert MANNA Düngerwerke GmbH, Nürnberg, Germany) in 1 L tap water] before watering up to the usual weight.

At T1, T2 and T3 (38, 46 and 54 dps, respectively), 10 plants of each treatment (F treatment only at T2 and T3) were harvested. Harvests were always performed one day after watering. Discs (about 160 mg fresh weight) from the youngest fully developed leaf were cut for glucosinolate analysis and determination of the specific leaf mass. From the next oldest leaf, discs (about 200 mg fresh weight) were cut for analysis of amino acids and the C:N ratio. Leaf discs were weighed, rapidly frozen in liquid nitrogen, stored at −80 °C and lyophilized. The remaining shoots were cut above the cotyledons, weighed and dried at 45 °C for three days. The dry mass of all samples was determined.

### 4.2. Determination of Water Content, Specific Leaf Mass, and Water Use Efficiency

The relative water content was determined from the fresh and dry biomass of the shoots. The specific leaf mass was determined by dividing the dry mass of the leaf discs by their area. The WUE_applied_ was determined based on [72], dividing the dry shoot biomass by the cumulative irrigation amount, a plant received over the experimental period. For the flooded plants, cumulative irrigation could not be determined.

### 4.3. Determination of C and N Contents, Amino Acids and Glucosinolates

Leaf samples were homogenized. About 3 (±0.1) mg (*n* = 10 per harvest time point and treatment) was taken for analysis of the C and N contents using a CN-analyzer (Elementar, Langenselbold, Germany).

Amino acid analysis was performed following the procedure in [73], with modifications. About 5 (±1) mg of leaf powder (*n* = 6 per harvest time point and treatment) was extracted three-fold in 80% methanol (LC-MS grade, Thermo Fisher Scientific, Waltham, MA, USA), adding norvaline and sarcosine (Agilent Technologies, Waldbronn, Germany) as internal standards for primary and secondary amino acids, respectively. Supernatants were pooled, filtered (0.2 µm syringe filters; Phenomenex, Torrance, CA, USA) and stored at −80 °C until analysis. Amino acids were analyzed via high-performance liquid chromatography (HPLC) coupled to fluorescence detection (1260/1290 Infinity, Agilent Technologies, Santa Clara, CA, USA) with a ZOR-BAX Eclipse Plus C 18 column (250 mm × 4.6 mm, 5 µm particle size, with guard column; Agilent Technologies). Samples were derivatized pre-column at 6 °C with borate-buffer (0.4 M, pH = 10.2; Agilent Technologies), ortho-phthaldialdehyde (OPA, 10 mg/mL in borate buffer and 3-mercapotpropionic acid; Agilent Technologies), 9-fluorenyl-methyl chloroformate (FMOC, 2.5 mg mL^−1^ in acetonitrile; Agilent Technologies) and an injection diluent [100 mL eluent A (see below) mixed with 0.4 mL 85% phosphoric acid (AppliChem GmbH, Darmstadt, Germany). A gradient of eluent A [1.4 gL^−1^ Na_2_HPO_4_ (Carl Roth GmbH + Co. KG, Karlsruhe, Germany), 3.8 g L^−1^ Na*2*B_4_0_7_ ∙ 10 H_2_O (Sigma-Aldrich, St. Louis, MO, USA), 32 mg L^−1^ NaN_3_ (Carl Roth GmbH + Co. KG) in Millipore water, pH = 8.2, and 85% phosphoric acid (AppliChem) in a ratio of 1:0.004 (*v*:*v*)] and eluent B [4.5 acetonitrile: 4.5 methanol (both LC-MS grade, Fisher Scientific): 1 demineralized water *v:v:v*] was used. The gradient started at 2% B for 0.84 min and increased within 53.4 min up to 57% B. The column was then rinsed and equilibrated before injection of the next sample. The flow rate was 1.5 mL min^−1^, and the column temperature was 40 °C. Amino acids derivatized with OPA or FMOC had an excitation wavelength of 340 nm or 260 nm, and were emitted at 450 nm or 325 nm, respectively. Amino acids were identified by the retention times of reference standards measured in the same batch and quantified by their peak heights, normalized by the respective internal standards and related to the dry mass of the sample. Amino acids were only included in the dataset if the mean of the amino acid in at least one group (per harvest time point and treatment) was at least five times higher than the mean of the blanks and if it occurred in at least 50% of the replicates.

For glucosinolate analysis, about 15 (±1) mg of leaf powder (*n* = 10 per treatment and harvest time point) were extracted threefold in 80% methanol, adding *p*-hydroxybenzyl glucosinolate (Phytoplan Diehm & Neuberger, Germany) as an internal standard. Supernatants were applied on anion exchange columns [Sephadex A25 (AppliChem, Darmstadt, Germany), in 0.5 M acetic acid buffer, pH = 5.0], and columns were washed with water and subsequently with 0.02 M acetic acid buffer (pH = 5.0). Finally, *Helix pomatia* sulfatase (Sigma-Aldrich; in 0.02 M acetic acid buffer), purified according to [74], was applied to the columns for the conversion of glucosinolates into desulfoglucosinolates. After one day, columns were washed with Millipore water, and solutions were dried and resolved in water. The desulfoglucosinolates were analyzed via HPLC (Dionex Ultimate 3000, Thermo Fisher Scientific, Waltham, MA, USA) coupled with a diode-array detector (210–370 nm) with a Supelcosil LC18, reversed phase (150 × 3 mm, 3 µm) column (Supelco, Bellefonte, PA, USA), at 25 °C column temperature. A gradient of eluent A (Millipore water) and eluent B (methanol) at a flow rate of 0.35 mL min^−1^ was used, starting at 5% B, held for 6.0 min, increasing to 38% B until 12.2 min, to 60% B until 14.3 min, to 60% until 16.6 min and to 90% B until 21.0 min, followed by a column cleaning and equilibration cycle. Glucosinolates were identified by comparing their retention times and spectra to an in-house databank. For quantification, peaks were related to those of the internal standard, applying the response factors 1, 0.5 and 0.26 for aliphatic, aromatic and indolic glucosinolates, respectively, and related to sample dry mass.

Fold changes were determined for amino acids and glucosinolates by dividing the mean concentration of one metabolite within one of the stress groups at one harvest time point by the corresponding mean of the metabolite in the control group at the same harvest time point. A metabolite did only remain in the dataset if it occurred in at least 50% of the replicates of one group. Groups of the fold changes as well as the metabolites were clustered with Cluster 3.0 [75] using average linkage hierarchical clustering and a Euclidian correlation distance matrix. Fold changes of the metabolites were presented in a heatmap using JavaTreeView [76]. Pathways of the metabolites with the corresponding color-codes of the heatmap were shown in a metabolic map based on [37] and [38]. Missing pathways were constructed using KEGG PATHWAY database [77]. Metabolites were considered to be “modulated” by the treatment based on two criteria, i.e., a highly significant difference between the stress treatment and control group (*p* < 0.01) and fold changes of <0.67 (−0.58 on log_2_ scale; lower concentration in stress group compared to control) or >1.5 (>0.58 on log_2_ scale; higher concentration in stress group).

### 4.4. Statistical Analysis

All statistical analyses were performed with RStudio [78] under R 4.0.3, using the packages *pgirmess, car* and *vegan*. Data were tested for normal distribution using the Shapiro–Wilk test and homoscedasticity using the Levene test. Accordingly, data from T1 (C vs. D) were analyzed with *t*-tests or Mann–Whitney *U*-tests (at T1). For the comparison of three groups (datasets at T2 and T3), univariate analysis of variance (ANOVA) followed by Tukey-HSD tests or Kruskal–Wallis-tests followed by Kruskal-mc post hoc tests were performed. To visualize the composition of amino acids and glucosinolates, respectively, non-metric multidimensional scaling (NMDS) with a Wisconsin double standardization of square-root transformed data was performed, using the Kulczynski distance. For the comparison of each of the two groups that were compared in the heatmap and the metabolic map, Mann–Whitney *U*-tests (without correction for multiple comparisons) were calculated.

## Figures and Tables

**Table 1:** Identified metabolites in the leaves of savoy cabbage (*Brassica oleracea* convar. *capitata* var. *sabauda*). The type of metabolite, as well as the used abbreviations and the retention time (Ret. time), are given.

Type	Metabolite	Abbreviation	Ret. Time
Primary amino acids	Aspartic acid	ASP	2.60
	Glutamic acid	GLU	4.48
	Asparagine	ASN	9.29
	Serine	SER	9.97
	Glutamine	GLN	11.92
	Histidine	HIS	12.60
	Threonine	THR	13.87
	Arginine	ARG	17.00
	Alanine	ALA	17.52
	*gamma*-Aminobutyric acid	GABA	18.33
	Tyrosine	TYR	21.69
	Methionine	MET	27.85
	Tryptophan	TRP	30.65
	Phenylalanine	PHE	31.70
	Isoleucine	ILE	32.20
	Leucine	LEU	34.25
	Lysine	LYS	35.98
Secondary amino acids	Hydroxyproline	HYP	37.59
	Proline	PRO	45.99
Glucosinolates (GLSs)	3-(Methylsulfinyl)propyl GLS	3MSOP	4.4
	4-(Methylsulfinyl)butyl GLS	4MSOB	6.7
	Allyl GLS	Allyl	7.6
	But-3-enyl GLS	3But	14.2
	4-Hydroxyindol-3-ylmethyl GLS	4OHI3M	14.4
	Indol-3-ylmethyl GLS	I3M	17.2
	4-Methoxyindol-3-ylmetyl GLS	4MOI3M	18.5
	1-Methoxyindol-3-ylmethyl GLS	1MOI3M	19.9

## Data Availability

The data of this study will be made available online via https://pub.uni-bielefeld.de/, accessed on 7 December 2021.

## References

[1] Rosenzweig C., Iglesias A., Yang X.B., Epstein P.R., Chivian E. (2001). Climate change and extreme weather events: Implications for food production, plant diseases, and pests. Glob. Chang. Hum. Health.

[2] Lehmann J., Coumou D., Frieler K. (2015). Increased record-breaking precipitation events under global warming. Clim. Chang..

[3] Hansen J., Sato M., Ruedy R. (2012). Perception of climate change. Proc. Natl. Acad. Sci. USA.

[4] Farooq M., Wahid A., Kobayashi N., Fujita D., Basra S.M.A. (2009). Plant drought stress: Effects, mechanisms and management. Agron. Sustain. Dev..

[5] Ahuja I., de Vos R.C.H., Bones A.M., Hall R.D. (2010). Plant molecular stress responses face climate change. Trends Plant Sci..

[6] Chaves M.M., Maroco J.P., Pereira J.S. (2003). Understanding plant responses to drought—From genes to the whole plant. Funct. Plant Biol..

[7] Irfan M., Hayat S., Hayat Q., Afroz S., Ahmad A. (2010). Physiological and biochemical changes in plants under waterlogging. Protoplasma.

[8] Bashar K.K., Tareq M.Z., Amin M.R., Honi U., Tahjib-Ul-Arif M., Abu Sadat M., Hossen Q.M.M. (2019). Phytohormone-mediated stomatal response, escape and quiescence strategies in plants under flooding stress. Agronomy.

[9] Kumutha D., Ezhilmathi K., Sairam R.K., Srivastava G.C., Deshmukh P.S., Meena R.C. (2009). Waterlogging induced oxidative stress and antioxidant activity in pigeonpea genotypes. Biol. Plant..

[10] Bodner G., Nakhforoosh A., Kaul H.P. (2015). Management of crop water under drought: A review. Agron. Sustain. Dev..

[11] Korres N.E., Norsworthy J.K., Tehranchian P., Gitsopoulos T.K., Loka D.A., Oosterhuis D.M., Gealy D.R., Moss S.R., Burgos N.R., Miller M.R. (2016). Cultivars to face climate change effects on crops and weeds: A review. Agron. Sustain. Dev..

[12] Qian L., Chen X.H., Wang X.G., Huang S., Luo Y.Y. (2020). The effects of flood, drought, and flood followed by drought on yield in cotton. Agronomy.

[13] Xiong Q.Q., Deng Y., Zhong L., He H.H., Chen X.R. (2018). Effects of drought-flood abrupt alternation on yield and physiological characteristics of rice. Int. J. Agric. Biol..

[14] González J.A., Gallardo M., Hilal M., Rosa M., Prado F.E. (2009). Physiological responses of quinoa (*Chenopodium quinoa* Willd.) to drought and waterlogging stresses: Dry matter partitioning. Bot. Stud..

[15] Husen A., Iqbal M., Aref I.M. (2014). Growth, water status, and leaf characteristics of *Brassica carinata* under drought and rehydration conditions. Braz. J. Bot..

[16] Nguyen L.T.T., Osanai Y., Anderson I.C., Bange M.P., Tissue D.T., Singh B.K. (2018). Flooding and prolonged drought have differential legacy impacts on soil nitrogen cycling, microbial communities and plant productivity. Plant Soil.

[17] Kang W., Kang S.J. (2019). On the use of alternative water use efficiency parameters in dryland ecosystems: A review. J. Ecol. Environ..

[18] Stallmann J., Schweiger R., Pons C.A.A., Müller C. (2020). Wheat growth, applied water use efficiency and flag leaf metabolome under continuous and pulsed deficit irrigation. Sci. Rep..

[19] Zegada-Lizarazu W., Iijima M. (2005). Deep root water uptake ability and water use efficiency of pearl millet in comparison to other millet species. Plant. Prod. Sci..

[20] Mony C., Mercier E., Bonis A., Bouzille J.B. (2010). Reproductive strategies may explain plant tolerance to inundation: A mesocosm experiment using six marsh species. Aquat. Bot..

[21] Ludewig K., Hanke J.M., Wuthe B., Otte A., Mosner E., Eckstein R.L., Donath T.W. (2018). Differential effect of drought regimes on the seedling performance of six floodplain grassland species. Plant Biol..

[22] Sardans J., Rivas-Ubach A., Penuelas J. (2012). The C:N:P stoichiometry of organisms and ecosystems in a changing world: A review and perspectives. Perspect. Plant Ecol. Evol. Syst..

[23] Lothier J., Diab H., Cukier C., Limami A.M., Tcherkez G. (2020). Metabolic responses to waterlogging differ between roots and shoots and reflect phloem transport alteration in *Medicago truncatula*. Plants.

[24] Ali Q., Athar H.U.R., Haider M.Z., Shahid S., Aslam N., Shehzad F., Naseem J., Ashraf R., Ali A., Hussain S.M., Hasanuzzaman M., Fujita M., Oku H., Islam M.T. (2019). Role of amino acids in improving abiotic stress tolerance to plants. Plant Tolerance to Environmental Stress.

[25] Orsák M., Kotíková Z., Hnilička F., Lachman J. (2021). Effect of drought and waterlogging on saccharides and amino acids content in potato tubers. Plant Soil Environ..

[26] Ashraf M., Mehmood S. (1990). Effects of waterlogging on growth and some physiological parameters of four *Brassica* species. Plant Soil.

[27] Hayat S., Hayat Q., Alyemeni M.N., Wani A.S., Pichtel J., Ahmad A. (2012). Role of proline under changing environments. A review. Plant Signal. Behav..

[28] Blažević I., Montaut S., Burčul F., Olsen C.E., Burow M., Rollin P., Agerbirk N. (2020). Glucosinolate structural diversity, identification, chemical synthesis and metabolism in plants. Phytochemistry.

[29] Bekaert M., Edger P.P., Hudson C.M., Pires J.C., Conant G.C. (2012). Metabolic and evolutionary costs of herbivory defense: Systems biology of glucosinolate synthesis. New Phytol..

[30] Knight H., Knight M.R. (2001). Abiotic stress signalling pathways: Specificity and cross-talk. Trends Plant Sci..

[31] Spoel S.H., van Ooijen G. (2014). Circadian redox signaling in plant immunity and abiotic stress. Antioxid. Redox Signal..

[32] Variyar P.S., Banerjee A., Akkarakaran J.J., Suprasanna P., Ahmad P., Rasool S. (2014). Role of glucosinolates in plant stress tolerance. Emerging Technologies and Management of Crop Stress Tolerance.

[33] Metz J., Ribbers K., Tielbörger K., Müller C. (2014). Long- and medium-term effects of aridity on the chemical defence of a widespread Brassicaceae in the Mediterranean. Environ. Exp. Bot..

[34] Burow M. (2016). Complex environments interact with plant development to shape glucosinolate profiles. Adv. Bot. Res..

[35] Schreiner M., Beyene B., Krumbein A., Stutzel H. (2009). Ontogenetic changes of 2-propenyl and 3-indolylmethyl glucosinolates in *Brassica carinata* leaves as affected by water supply. J. Agric. Food Chem..

[36] Sasidharan R., Bailey-Serres J., Ashikari M., Atwell B.J., Colmer T.D., Fagerstedt K., Fukao T., Geigenberger P., Hebelstrup K.H., Hill R.D. (2017). Community recommendations on terminology and procedures used in flooding and low oxygen stress research. New Phytol..

[37] Schweiger R., Baier M.C., Persicke M., Müller C. (2014). High specificity in plant metabolic responses to arbuscular mycorrhiza. Nature Commun..

[38] Bonte A., Schweiger R., Pons C., Wagner C., Brühl L., Matthäus B., Müller C. (2017). Metabolic changes during storage of *Brassica napus* seeds under moist conditions and consequences for the sensory quality of the resulting virgin oil. J. Agric. Food Chem..

[39] Osakabe Y., Osakabe K., Shinozaki K., Tran L.S.P. (2014). Response of plants to water stress. Front. Plant Sci..

[40] Sanoubar R., Cellini A., Veroni A.M., Spinelli F., Masia A., Antisari L.V., Orsini F., Gianquinto G. (2016). Salinity thresholds and genotypic variability of cabbage (*Brassica oleracea* L.) grown under saline stress. J. Sci. Food Agric..

[41] Ashraf M., Mehmood S. (1990). Response of four *Brassica* species to drought stress. Environ. Exp. Bot..

[42] Ku Y.-G., Park W., Bang J.-K., Jang Y.-S., Kim Y.-B., Bae H.-J., Suh M.-C., Ahn S.-J. (2009). Physiological response, fatty acid composition and yield component of *Brassica napus* L. under short-term waterlogging. J. Bio-Environ. Control.

[43] Scandalios J.G. (1993). Oxygen stress and superoxide dismutases. Plant Physiol..

[44] Van Dongen J.T., Licausi F. (2015). Oxygen sensing and signaling. Annu. Rev. Plant Biol..

[45] Voesenek L., Bailey-Serres J. (2015). Flood adaptive traits and processes: An overview. New Phytol..

[46] Ren B.Z., Zhang J.W., Dong S.T., Liu P., Zhao B. (2016). Effects of waterlogging on leaf mesophyll cell ultrastructure and photosynthetic characteristics of summer maize. PLoS ONE.

[47] Chang R., Jang C.J.H., Branco-Price C., Nghiem P., Bailey-Serres J. (2012). Transient MPK6 activation in response to oxygen deprivation and reoxygenation is mediated by mitochondria and aids seedling survival in *Arabidopsis*. Plant Mol. Biol..

[48] Savchenko T., Rolletschek H., Heinzel N., Tikhonov K., Dehesh K. (2019). Waterlogging tolerance rendered by oxylipin-mediated metabolic reprogramming in Arabidopsis. J. Exp. Bot..

[49] Wollmer A.C., Pitann B., Muhling K.H. (2018). Waterlogging events during stem elongation or flowering affect yield of oilseed rape (*Brassica napus* L.) but not seed quality. J. Agro. Crop Sci..

[50] Strohm K., Garming H., Dirksmeyer W. (2016). Entwicklung des Gemüsebaus in Deutschland von 2000 bis 2015: Anbauregionen, Betriebsstrukturen, Gemüsearten und Handel.

[51] Zhu M., Monroe J.G., Suhail Y., Villiers F., Mullen J., Pater D., Hauser F., Jeon B.W., Bader J.S., Kwak J.M. (2016). Molecular and systems approaches towards drought-tolerant canola crops. New Phytol..

[52] Tewes L.J., Müller C. (2018). Syndromes in suites of correlated traits suggest multiple mechanisms facilitating invasion in a plant range-expander. NeoBiota.

[53] Buttar G.S., Thind H.S., Aujla M.S. (2006). Methods of planting and irrigation at various levels of nitrogen affect the seed yield and water use efficiency in transplanted oilseed rape (*Brassica napus* L.). Agric. Water Manag..

[54] Franco J.A., Bañón S., Vicente M.J., Miralles J., Martínez-Sánchez J.J. (2011). Root development in horticultural plants grown under abiotic stress conditions—A review. J. Hortic. Sci. Biotech..

[55] Fry E.L., Evans A.L., Sturrock C.J., Bullock J.M., Bardgett R.D. (2018). Root architecture governs plasticity in response to drought. Plant Soil.

[56] Alam R., Iqbal A., Khan I., Ali I., Munir I., Tahir M., Jan N., Swati Z.A. (2011). Carbon and nitrogen stoichiometry in *Brassica napus* L. seedlings after supplementation with Ca^2+^ and K^+^ under irrigated and drought stress conditions. Afr. J. Biotechnol..

[57] Butt M., Ayyub C.M., Amjad M., Ahmad R. (2016). Proline application enhances growth of chilli by improving physiological and biochemical attributes under salt stress. Pak. J. Agric. Sci..

[58] Ullah F., Bano A., Nosheen A. (2012). Eeffects of plant growth regulators on growth and oil quality of canola (*Brassica napus* L.) under drought stress. Pak. J. Bot..

[59] Pons C., Voß A.-C., Schweiger R., Müller C. (2020). Effects of drought and mycorrhiza on wheat and aphid infestation. Ecol. Evol..

[60] An Y.Y., Zhang M.X., Liu G.B., Han R.L., Liang Z.S. (2013). Proline accumulation in leaves of *Periploca sepium* via both biosynthesis up-regulation and transport during recovery from severe drought. PLoS ONE.

[61] Kavi Kishor P.B.K., Sangam S., Amrutha R.N., Laxmi P.S., Naidu K.R., Rao K.R.S.S., Rao S., Reddy K.J., Theriappan P., Sreenivasulu N. (2005). Regulation of proline biosynthesis, degradation, uptake and transport in higher plants: Its implications in plant growth and abiotic stress tolerance. Curr. Sci..

[62] Szabados L., Savouré A. (2010). Proline: A multifunctional amino acid. Trends Plant Sci..

[63] Kim Y., Seo C.W., Khan A.L., Mun B.G., Shahzad R., Ko J.W., Yun B.W., Park S.K., Lee I.J. (2018). Exo-ethylene application mitigates waterlogging stress in soybean (*Glycine max* L.). BMC Plant Biol..

[64] Fischer M., Kaldenhoff R. (2008). On the pH regulation of plant aquaporins. J. Biol. Chem..

[65] Frick A., Järvå M., Törnroth-Horsefield S. (2013). Structural basis for pH gating of plant aquaporins. FEBS Lett..

[66] Moustafa-Farag M., Mahmoud A., Arnao M.B., Sheteiwy M.S., Dafea M., Soltan M., Elkelish A., Hasanuzzaman M., Ai S.Y. (2020). Melatonin-induced water stress tolerance in plants: Recent advances. Antioxidants.

[67] Tsai K.J., Lin C.Y., Ting C.Y., Shih M.C. (2016). Ethylene-regulated glutamate dehydrogenase fine-tunes metabolism during anoxia-reoxygenation. Plant Physiol..

[68] Khan M.A.M., Ulrichs C., Mewis I. (2011). Water stress alters aphid-induced glucosinolate response in *Brassica oleracea* var. *italica* differently. Chemoecology.

[69] Kuczyk J., Müller C., Fischer K. (2021). Plant-mediated indirect effects of climate change on an insect herbivore. Basic Appl. Ecol..

[70] Tariq M., Rossiter J.T., Wright D.J., Staley J.T. (2013). Drought alters interactions between root and foliar herbivores. Oecologia.

[71] ISO 11465 (1993). Soil Quality—Determination of Dry Matter and Water Content on a Mass Basis—Gravimetric Method.

[72] Boyle R.K.A., McAinsh M., Dodd I.C. (2016). Stomatal closure of *Pelargonium x hortorum* in response to soil water deficit is associated with decreased leaf water potential only under rapid soil drying. Physiol. Plant..

[73] Jakobs R., Müller C. (2018). Effects of intraspecific and intra-individual differences in plant quality on preference and performance of monophagous aphid species. Oecologia.

[74] Graser G., Oldham N.J., Brown P.D., Temp U., Gershenzon J. (2001). The biosynthesis of benzoic acid glucosinolate esters in *Arabidopsis thaliana*. Phytochemistry.

[75] De Hoon M.J.L., Imoto S., Nolan J., Miyano S. (2004). Open source clustering software. Bioinformatics.

[76] Saldanha A.J. (2004). Java Treeview-extensible visualization of microarray data. Bioinformatics.

[77] Kanehisa M., Goto S. (2000). KEGG: Kyoto Encyclopedia of Genes and Genomes. Nucleic Acids Res..

[78] RStudio Team (2019). RStudio: Integrated Development for R.

